# Adipose-Derived Stem Cells for the Treatment of Diabetic Wound: From Basic Study to Clinical Application

**DOI:** 10.3389/fendo.2022.882469

**Published:** 2022-07-11

**Authors:** Runzhu Liu, Ruijia Dong, Mengling Chang, Xiao Liang, Hayson Chenyu Wang

**Affiliations:** ^1^ Department of Plastic and Reconstructive Surgery, Shanghai Ninth People’s Hospital, Shanghai Jiaotong University School of Medicine, Shanghai, China; ^2^ Peking Union Medical College and Chinese Academy of Medical Sciences, Beijing, China; ^3^ Department of Plastic Surgery, Beijing Tsinghua Changgung Hospital, School of Clinical Medicine, Tsinghua University, Beijing, China

**Keywords:** diabetic wound, adipose-derived stem cells (ASC), cell therapy, diabetic ulcer, diabetic wound healing

## Abstract

Diabetic wounds significantly affect the life quality of patients and may cause amputation and mortality if poorly managed. Recently, a wide range of cell-based methods has emerged as novel therapeutic methods in treating diabetic wounds. Adipose-derived stem cells (ASCs) are considered to have the potential for widespread clinical application of diabetic wounds treatment in the future. This review summarized the mechanisms of ASCs to promote diabetic wound healing, including the promotion of immunomodulation, neovascularization, and fibro synthesis. We also review the current progress and limitations of clinical studies using ASCs to intervene in diabetic wound healing. New methods of ASC delivery have been raised in recent years to provide a standardized and convenient use of ASCs.

## Introduction

The diabetic wound is a severe chronic complication of diabetes, which greatly affects the life quality of patients. Diabetic foot ulcer (DFU), as one of the most severe forms of diabetic wounds, is troubling about 6.3% population globally (1). The prominent manifestations include peripheral neuropathy and ischemia of the lower extremities, resulting in disturbance of sensation, muscle atrophy, resting pain, and gangrene which significantly reduce the quality of life (2). Furthermore, diabetic wounds could cause severe morbidity, amputation, and mortality in patients if the diabetic wound was not properly managed (3). Diabetic ulcers amputation is associated with a higher rate of infection, even bringing a higher medical financial burden than cardiac emergencies (4).

The occurrence of diabetic wounds derived from peripheral neuropathy contributes to the injury of flexibility, protective sensation, and secretory function of the skin, which results in a higher possibility of diabetic patients developing wounds on the skin (4). At the cellular level, diabetes damages the function of macrophages and prevents keratinocytes and fibroblasts’ functions in epithelial healing (5, 6). Hyperglycemia causes endothelial injury of the peripheral vascular, which further reduces the skin perfusion, promoting the formation of a cutaneous ulcer (5). Oxygen deficit, reduced vascular perfusion, elevated oxidative stress, and superimposing of infection are the main difficulties in diabetic wound treatment (7). The management of diabetic wounds is based on principles raised by Dr Frederick Treves, who advocated surgical debridement, wound off-loading, and blood glucose control as standard care for diabetic wounds (8). However, the development of diabetic wounds is always urgent and non-intervention treatment is incapable to control wound progression. Over the past decades, few efficient treatments for diabetic wounds were applied because of the lack of targeted therapy (9).

Recently, a wide range of cell-based methods has emerged as novel therapeutic methods in treating diabetic wounds, with evidence showing that the transplantation of keratinocytes, fibroblasts, endothelial progenitor cells (EPCs), mesenchymal stem cells (MSCs) may benefit diabetic wound healing (10, 11). These cells are component of epidermal and important sources of cytokines, chemokines, and growth factors essential in wound healing. Apart from the component cells, stem-cell therapies also showed considerable promise on chronic diabetic wound healing without surgical complications and donor site injuries (12), which are classified into allogenic and autologous cells (9). Amnion-derived MSCs and embryonic stem cells (ESCs) have been used as allogeneic stem cell treatment in diabetic wounds (9). Bone-marrow derived MSCs (BM-MSCs), bone-marrow derived endothelial progenitor cells (EPCs), hematopoietic stem cells (HSCs) and adipose-derived stem cells (ASCs) can be isolated from autologous tissues, which are potentially benefit on diabetic wound treatment (9). Further characteristics of these cells are shown in **Table 1**. In previous studies, BM-MSCs are focused because of the therapeutic effect being showed in both animal studies and clinical studies (26, 27). ASCs, the most accessible source of MSCs, are considered to have the potential for widespread clinical application of diabetic wounds treatment in the future (17). This review focuses on the therapeutic effects of ASCs, aiming to summarize the current progress and limitations on ASCs in diabetic wounds treatment.

**Table 1:** A brief comparison of characteristics of different types of stem cells.

	Main mechanism	Source	Strength	Limitations
Allogenic MSCs	vasculogenesis (13)	Placenta (14), umbilical cord (15), amniotic fluid (16);	Easy access; Hypoimmunogenic; Source of pluripotent cells; Noninvasive (13); Lower immunogenicity (17);	Risk of tumorigenicity; Risk of immunogenicity; Lack of orientation (9);
ESCs	Endocrine growth factors, vasculogenesis;	Inner layer cell of *in vitro* embryos (9)	Able to differentiate into any cell line (9);	Controversial source from *in vitro* embryos (9); Technically difficult to generate; Immune rejection (18);
BM-MSCs	Vasculogenesis; Fibrogenesis; Immunomodulation;	Bone marrow	Clear benefits to diabetic wounds;	Invasive; Costly; Strict culture environment; Less immune compacity (19);
iPSCs	Compensate for lack of epidermal component (18);	Reprogramming differentiated cells (18);	Ease to access; Abundant (18); Less ethical concern;	Critical cell culture condition; Tumorigenicity (18); Immune rejection; High cost;
HSCs	Immunomodulation; Promotion of cell proliferation (20);	Bone marrow, umbilical cord; Peripheral blood (21);	Ease of access;	Limited differentiation potential;
EPCs	Angiogenesis; Response to tissue damage and ischemia (22);	Bone marrow;	Potential of treatment in pathological conditions (11);	Few therapeutic evidence; Need reprograming; Need homing factors (11);
ASCs	Vasculogenesis; Fibrogenesis; Immunomodulation;	Adipose tissue	Fewer ethical problem; high colony frequency (23); less invasive; Source of pluripotent cells (24);	Donor-site morbidity (25);

(MSCs, mesenchymal stem cells; ESCs, embryonic stem cells; BM-MSCs, Bone-marrow derived MSCs; iPSCs, Induced pluripotent stem cells; HSCs, hematopoietic stem cells; EPCs, endothelial progenitor cells; ASCs, adipose-derived stem cells).

## Mechanisms of ASCs to Promote Diabetic Wound Healing

**Figure 1:**
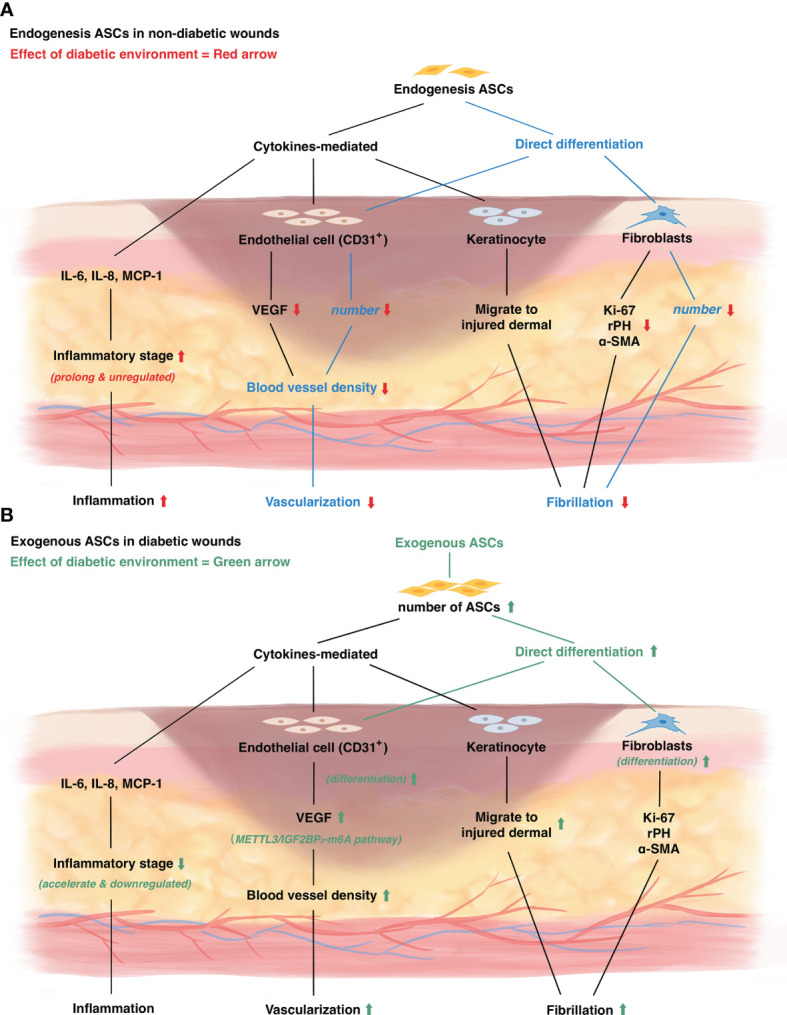
The mechanism of diabetic wound healing and the role of adipose derived stem cells in diabetic wounds. **(A)** The change of endogenous ASCs in diabetic wounds. Red arrow represents diabetic environment influence on wound healing. **(B)** The influence of exogenous ASCs on diabetic wounds. Green arrow represents the mechanism of exogenous ASCs treatment on diabetic wound healing.

ASCs are convenient to access as they can be derived from autologous tissues or allogenic tissues in the abdominal region, inguinal region, and thigh from liposuction (28). ASCs have similar characteristics in self-renewal, differentiation, and proliferation to MSCs (29). ASCs can differentiate into adipocytes, endothelial cells, fibroblasts, and other cells. ASCs have significant potential in fibroblastic morphology than other MSCs sources, which plays an essential role in dermal remodeling (30). They are also found to differentiate into endothelial cells and promote vascularization (30). ASCs are normal components in healthy individuals and work as important roles in wound healing. In response to injury, ASCs represent both paracrine function and the ability to differentiate directly into epithelial components (30). ASCs are shown to secret various cytokines such as vascular endothelial growth factor (VEGF), fibroblast growth factor 2 (FGF2), keratinocyte growth factor (KGF), transforming growth factor-beta (TGF-β), platelet-derived growth factor (PDGF), hepatocyte growth factor (HGF) and collagen (31). Animal studies and pre-clinical studies are reviewed to predict the role of ASCs in diabetic wound healing. The detailed information of included studies is shown in **Table 2**. The specific mechanism of ASCs functions in diabetic wound healing is shown in **Figure 1**.

**Table 2:** Summary of ASCs treatment on diabetic wounds in animal models.

Authors, year	Models/Species	Source	Dosage(cells/wound)	Route	Time	Main result
2011 Kim, et al. (32)	STZ 150mg/kg, i.p. induced rats (DM1)	Human ASCs	3×10^6^	peri-wound injection	24h after surgery	Gross morphology, histology, tissue VEGF
2011 Lee, et al. (33)	ketamine (75 mg/kg) and xylazine (15 mg/kg) induced rats	Human ASCs	NA	Direct cover on the wound	Immediately after surgery	wound size, histology of wounds
2011 Nie, et al. (34)	STZ 65mg/kg, i.p. induced rats (DM1)	Allogenic ASCs	1×10^6^	peri-wound injection	Immediately after surgery	wound closure area, histology of wounds, vessel density, immunofluorescent analysis
2014 Cianfarani, et al. (35)	STZ 40mg/kg, i.p. induced rats (DM1)	Allogenic SVF cells	5×10^5^	Direct spray over wound	Immediately after surgery	Cytokine levels, cell amounts, granulation tissue area, veseel density
2015 Kato, et al. (36)	ZDF-Leprfa/CrlCrlj (DM2)	Allogenic ASCs	NA	cell sheet cover on the wound	NA	Wound closure time, Blood vessel densities, fate of transplanted ASCs
2016 Kuo, et al. (37)	STZ 50mg/kg, i.p. induced rats (DM1)	Allogenic ASCs	1×10^7^	peri-wound injection	NA	wound size, peri wound inflammatory responses, fate of transplanted ASCs
2016 Massee, et al. (38)	human ASCs. T2DM&T1DM	Human ASCs	NA	–	NA	proliferation and migration ability of ASCs, secretion function of ASCs
2016 Shi, et al. (39)	STZ 100mg/kg, i.p. induced rats (DM1)	Human ASCs	5×10^6^	peri-wound injection	Immediately after surgery	ulceration contraction rate, histology assessment, vessel density
2017 Hamada, et al. (40)	ZDF-Leprfa/CrlCrlj (DM2)	Human ASCs	7×10^5^	peri-wound injection	NA	Wound area, histological analysis of wound
2017 Kaisang, et al. (41)	STZ 50mg/kg, i.p. induced rats (DM1)	Allogenic ASCs	1×10^6^	Topical gel	NA	Percentage of wound closure, histology, blood vessel density, cytokine level
2017 Lin, et al. (41)	STZ 50mg/kg, i.p. induced rats (DM1)	Allogenic ASCs	1×10^6^	Topical gel	Immediately after surgery	wound closure, histology of wound, blood vessel density, expression of growth factors
2017 Seo, et al. (42)	Diabetic db/db mice	Human ASCs	2.5 × 10^5^	peri-wound injection	24h after surgery	Wound healing rate, histology of wound skin, cytokines expression
2018 Irons, et al. (43)	STZ 150mg/kg i.p. induced Yorkshire swine	Allogenic ASCs	5×10^6^,10×10^6^	Peri-wound injection	Every 12h for the first 72h	Wound closure, histological analysis, mRNA and protein analyses
2018 Zhou, et al. (44)	alloxan infusion (130 mg/kg, Sigma–Aldrich) induced rabbit	Human ASCs	NA	peri-wound injection	7 days after surgery	wound area, histology of tissue healing, changes in cytokine factors
2019 Chen, et al. (45)	STZ 35mg/kg, i.p. induced rats (DM1)	Autologous nanofat	2×10^5^	Peri-wound injection	Immediately after surgery	Wound area change, vessel density, angiogenic factor expression
2019 Liu, et al. (46)	0.1 ml/10 g chloral hydrate induced rats,	Human ASCs	1×10^6^	peri-wound injection	2 days after surgery	wound healing rate, histology of wounds, immunohistochemical assay
2020 Ahmadi, et al. (47)	STZ 40mg/kg, i.p. induced rats (DM1)	Allogenic PBM treated ASCs	2×10^7^	Topical gel	Immediately after surgery	Wound closure rate, cell amount in the peri-wound area
2020 An, et al. (48)	STZ 180mg/kg, i.p. induced rats (DM1)	Allogenic normal ASCs	5×10^5^	peri-wound injection	NA	Cell type after injection, cytokine level, wound closure rate
2020 Ding, et al. (49)	STZ 165mg/kg, i.p. induced rats (DM1)	Allogenic Bcl-2-modified ASCs	NA	Direct spray over wound	7 days after surgery	Healing rate, histology, vascularization
2021 Ahmadi, et al. (50)	STZ 40mg/kg, i.p. induced rats (DM1)	Allogenic PBM treated ASCs	1×10^6^	peri-wound injection	NA	Wound maximum force, mast cell numbers, wound healing rate
2021 Ebrahim, et al. (51)	STZ 40mg/kg, i.p. induced rats (DM1)	Allogenic PRP+ASCs	2×10^6^	peri-wound injection	Immediately after surgery	Wound area, histological analysis, epidermal thickness, dermal collagen, angiogenesis.
2021 Laiva, et al. (52)	Human ADSCs iXCells Biotechnologies	Human ASCs	NA	–	NA	Expression of functional factors
2021 Zhou, et al. (53)	STZ 150mg/kg, i.p. induced rats (DM1)	Allogenic ASCs	400,000 cells/cm^2^	cell sheet cover on the wound	immediately after surgery	wound healing rate, histology of wounds

(STZ, streptozotocin; PBM, photobiomodulation; DM, Diabetes Mellitus; NA, not available).

### ASCs Act as Immunomodulators in Diabetic Wound Models

ASCs can downregulate the level of inflammatory cytokines and accelerate the progress of the inflammatory stage. A diabetic environment often leads to increased activity of inflammatory factors. Regeneration of injuries could be arrested under an inflammatory environment and lead to further injuries of peripheral tissues (54). The continuous state of inflammation leads to a higher level of inflammatory cytokines (such as IL-6, IL-8, monocyte chemotactic protein-1 MCP-1), as well as the abnormal presence of inflammatory cells (such as macrophages, monocytes, and neutrophils) (54). In previous studies, ASCs are inferred to influence the procedure of inflammation in chronic wounds by paracrine effects to suppress inflammation (30). To further verify the opinion, several studies evaluated the inflammatory cytokine variation in rat ASCs treated diabetic wounds (45, 48, 52). Cytokines such as IL-6, IL-8 are significantly deregulated in diabetic and non-diabetic ASCs treated wounds. Healthy ASCs result in a significantly lower level of cytokines than diabetic ASCs (38).

Meanwhile, MCP-1 expression is evaluated in the study of Chen et. al, in which autologous nano-fat transplantation of diabetic animal model was used (45). The evolution of MCP-1 levels in the nano-fat treated group is significantly lower than the controlled group, reaching a stable low expression seven days after injury, while the controlled group reached the low level of expression by the end of day 14 (45). ASCs can build a specific cell environment that down-regulated immune factors such as TNF-α, while the histologic examination confirmed more occasional giant cell inflammation in ASC-treated skin (43). Furthermore, this immune acceleration relies on the paracrine function of ASCs. In a study conducted by Laiva et. al, the application of SDF-1α scaffold, which promotes ASCs proliferation, showed no significant effect on high inflammatory cytokine caused by diabetic ASCs, indicating ASCs do not function as inflammatory cytokine secretion (52). In pre-clinical studies, human ASCs are used in diabetic wounds. As IL-6, IL-8, MCP-1 are upregulated in diabetic compared to healthy ASCs, and no evidence of expression changes after being treated with dHACM, it is further indicated that the effect of ASCs on inflammatory cytokinesis is not related to the ability to migration and proliferation of ASCs (38).

To further understand whether the function of ASCs is affected by the diabetic environment in immune-modulating, histological observation is also conducted in several studies using rat ASCs (37, 45, 47, 50, 51) and several studies using human ASCs (32, 46). It is shown that utilization of either diabetic or healthy ASCs on diabetic wounds reduces inflammatory cells infiltration. Diabetic ASCs treated wounds stay in an acute inflammatory stage, while healthy ASCs treated wounds and non-diabetic wounds have already switched into non-inflammatory stages (32). This suggests that non-diabetic ASCs can significantly accelerate the immune process more than diabetic wounds. In a study conducted by Ahmadi et.al, photo-biomodulation is introduced to diabetic ASCs under the condition of infectious diabetic wounds (50). This method significantly accelerated the switch from the inflammatory phase to the post-inflammatory phase within the use of diabetic ASCs.

### ASCs Promote Neovascularization in Diabetic Wound Models

ASCs are believed to take part in neovascularization by paracrine function and differentiation into endothelial cells. In healthy wounds, tissue injuries are followed by the production of pro-angiogenic factors, and angiogenesis is induced, which is impacted by a diabetic environment (55). The effect of ASCs on angiogenesis is evaluated through blood vessel densities by HE-staining or immunofluorescent labeling, endothelial cell densities by CD31+ expression, and pro-angiogenesis factors (40).

ASCs migrate to the margin of the diabetic wound and differentiate into endothelial cells to form new vessels. In general, blood vessel densities can be seen significantly increased in wounds treated with ASCs than in non-treated wounds (35, 36, 41, 41, 51). By immunofluorescence localization, newly formed vessel density in ASC-treated tissues is quantified as 2.5-folds higher than non-treated groups (51). The vessel density can be further elevated by the combined use of vehicles such as a bio-modulated gel (41, 41). Additionally, PRP can also promote the effect of ASCs (51), which may be used in making up the defects of diabetic ASCs. Immunofluorescence observational studies showed ASCs appear by the site where new vessels are formed (36, 45). Further exploration is achieved by immunofluorescence tracing the fate of injected ASCs (37, 48). It is shown that injected ASCs can migrate to the diabetic wound’s subdermal margin and differentiate directly into the vascular endothelial cells. In human ASCs studies, blood vessel densities and CD31^+^ cell amounts increase in healthy ASCs, treated wounds (32, 39, 41, 46, 49). Further studies show utilization of bio-modulated gel (41) and Bcl-2, which significantly increase the proliferation of ASCs, can further increase the vessel density in diabetic wounds (49). However, none of the studies evaluated whether the pro-vascularization function is decreased in diabetic human ASCs. Overall, by tracing the ASCs that are injected into the subdermal of wounds, it is confirmed that ASCs can migrate and differentiate into endothelial cells to participate in new vessel formation, and this process can be promoted by appropriate vehicles.

ASCs can also promote neovascularization by paracrine functions. Some growth factors that are related to angiogenesis were evaluated in some studies. As the most important and relevant growth factor in angiogenesis, the level of VEGF is reported to be upregulated in rat ASCs studies (34, 37, 41, 42, 45, 53, 56). It is reported that the level of VEGF, as well as TGF-β, is upregulated in granulations, while it can be further increased by combining the use of bio-modified gel (41). Further studies found the expression of VEGF is significantly higher in the tissue around injected ASCs (37). This may attribute to the enhancement of endothelial cell secretion function by ASCs. The molecular mechanism of this process is explored by Zhou et al. in 2021, indicating endothelial cell is promoted by ASCs to migration and forming tubule *via* VEGF-C-mediated METTL3/IGF2BP2−m6A pathway in diabetic mice (53). Studies using human ASCs also found a significantly higher level of VEGF in the tissue around the wound, no matter ASCs are harvested from diabetic or healthy individuals (32, 39, 42). Compared with a known angiogenesis factor, Exendin-4 (Ex-4), ASCs utilized from humans showed higher ability in promoting VEGF secretion by keratinocytes, and exhibit the ability to promote keratinocyte migration (42), confirming the hypothesis that ASCs can promote neovascularization by their paracrine function. Furthermore, in a comparative study, ASCs were found to connect with multiple pathways involved in angiogenesis, while BM-MSCs mainly correlated to cell adhesion and metabolic in diabetic wound treatment (57).

ASCs promote neovascularization by directly differentiating into endothelial cells and paracrine function to promote growth factors secretion in tissues. The regeneration of peripheral perfusion is essential in diabetic wound healing, determining the prognosis and recrudescence after healing. However, the ability of diabetic ASCs is not evaluated deeply in recent studies. As the most convenient source of ASC, it may be an important future task to explore the feature of diabetic ASCs in vascularization.

### ASCs Promote Fibro Synthesis in Diabetic Wound Models

ASCs participate in the process of fibrosis and epithelialization by direct differentiating into the components and paracrine effect on dermal fibroblasts and keratinocytes (30). In diabetic patients, the FGF and KI-67 expression represents cell proliferation potential and are limited in wound sites than in normal individuals (58). Diabetes can result in multiple dysfunctions which result in impaired function and the amount of peripheral ASCs around the wounds (59). ASCs promote fibrosis by increasing the secretion of fibroblast and keratinocytes as well as differentiate directly into fibroblasts, resulting in dermal collagen deposition and incrassation of granulation (30). Several studies looked into the influence of the diabetic environment on ASCs and how ASCs promote fibrosis in diabetic animals’ wounds.

In general, ASCs can promote fibrosis and fiber deposition to promote re-epithelialization in diabetic wounds. The thickness of dermal or granulation represents the extent of fibrosis and the end effect of ASCs on epithelialization. The thickness of the dermal layer is significantly increased in non-diabetic rat ASCs treated wounds (33–36, 41). To prove the superiority of ASCs over normal fibroblasts, the influence of ASCs on the thickening dermal layer was compared with the use of dermal fibroblasts (33, 34). The dermal layer is found to be thicker in both animal and human-derived ASCs treated wounds than fibroblasts-treated wounds, indicating ASCs have a higher effect of promoting epithelialization than dermal fibroblasts (47, 48). This result is observed by the end of 14 days after injuries (36, 48), which is around the stage of remodeling. The amounts of fibroblasts were quantified in some studies to evaluate the promotion of ASCs on fibroblasts, revealing an increment of fibroblasts in peri-wound tissues after ASCs injection (35, 47). By immunofluorescence labeling, ASCs are found to differentiate into fibroblasts after two weeks of injection (48). This revealed the potential of ASCs to promote epithelialization by differentiating directly into fibroblasts.

Besides, ASCs also have a paracrine function to promote fibroblasts and keratinocytes to produce more stroma, and to form epithelial ground substances. The formation of collagen was evaluated under HE-staining and observed under microscopes (47, 51, 52). Diabetic wounds showed thin and sparse collagen deposition than normal wounds, while ASCs treated wounds showed similar densities and thick bundles of collagen well arranged (51, 52). ASCs are also shown to induce the migration of fibroblasts and keratinocytes (57, 60). The proliferation and migration of epidermal-composed cells is related to multiple cell factors. Ki-67 expression was recognized to be elevated in ASCs treated tissues (37, 41). Other growth factors such as rPH, α-SMA expressed by fibroblasts are also detected to upregulated in ASCs treated wounds (37, 48). These results represent the paracrine function of rat ASCs on diabetic wounds. Furthermore, collagen deposition was reduced in diabetic ASCs treated wounds than healthy ASCs treated wounds (35), indicating a reduced effect of diabetic ASCs on paracrine function. Pre-clinical studies using human ASCs on diabetic animal rat wounds reached a similar conclusion (33, 39, 46). PRP utilization can further promote peri-wound collagen deposition in diabetic rats (46). Therefore, studies above showed healthy ASCs promote fibroblasts and keratinocytes to excrete more growth factors that respond to fibrosis and epithelialization.

### Effects of Diabetes on Autologous ASCs

In diabetic patients, autologous ASCs are weakened due to the hyperglycemia environment. Diabetic ASCs treated diabetic wounds are shown to have a longer time of complete healing than healthy ASCs treated wounds, and shorter than non-treated wounds (37, 48). Application of non-diabetic human-derived ASCs on diabetic rats showed the average time of wound closure and the healing area measured at the same time is significantly increased in normal ASCs treated rats than diabetic ASCs treated rats (32, 33, 46, 49, 61). Most studies evaluate the area of healing on 3, 7, 14 days after injuries. No significant differences are detected by the end of the 3-day follow-up, while all studies showed a significantly smaller wound area in ASCs treated rats by the end of the 7-day follow-up. Studies also showed that autocrine functions are significantly weakened in diabetic rat ASCs (35, 48). Diabetic rat ASCs are shown to express less VEGF-A, HGF, and IGF than healthy rat ASCs. VEGF-A has been proven to significantly ameliorate wound healing procedures by recruiting BM-MSCs to accelerate repairing in diabetes animal models (6, 62, 63). Although ASCs of both diabetic and healthy rats promote the proliferation and migration of fibroblasts and keratinocytes *in vitro* (35, 48), non-diabetic ASCs showed a better promotion in this process significantly than diabetic ASCs (35). Cianfarani et al. further evaluated the different features of ASCs between healthy and diabetic rats, in which diabetic ASCs showed the 0.6-folded ability of self-migration and proliferation than healthy ASCs (35). Furthermore, diabetic human ASCs are also being proved to secret a lower level of growth factors and a higher level of inflammatory factors than healthy human ASCs, which weakened the promotion in the wound healing process (38). Therefore, functions of ASCs in diabetic wounds are significantly decreased than normal ASCs, which may result in a poor healing pattern and may need a more exogenous complement.

## Clinical Progress of ASCs in Treating Diabetic Wounds

Based on the support of several pre-clinical experiments completed in animal models, some studies attempt to apply ASCs to clinical diabetes wound treatments in recent years. Several pilot studies (64–66) and randomized controlled trials (67, 68) were identified through databases. The detailed information of included clinical studies is shown in **Table 3**.

**Table 3:** Summary of clinical trials using ADCs to treat diabetic wounds. (SVF, stromal vascular fraction).

Authors, years	Treatment type	Cells/person	Wound site	Patient number	Target	Clinical outcome	Adverse events
2021 Carstens, et al. (64)	Autologous SVF cells injection	30×10^6^	lower extremity	59	Nonhealing diabetic ulcers, ≥3cm^2^, high risk of amputation	Ulcer size, time to closure, vessel flows, arterial wall elasticity	none
2019 Moon, et al. (65)	Autologous SVF cells injection	3.6 ± 0.2×10^7^	lower extremity	10	type I/II DM, TcPO2<40 mmHg, high risk of amputation	TcPO2 value change, cutaneous microvascular blood flow levels	none
2019 Moon, et al. (67)	Allogenic ADCs hydrogel	NA	lower extremity	59	type I/II DM, 1-25 cm^2^, Wagner grade I and II	complete wound closure percentage, mean time required for wound closure	None related to study dressing
2020 Nilforoushzadeh, et al. (66)	SVF-based full-thickness dermal cell grafts	NA	Lower extremity	10	Full-thickness neuropathic ulcer, >3 weeks	Wound area change percentage, dermal and epidermal thickness and density	None
2021Uzun, et al. (68)	Allogenic ASCs injection	6×10^6^	Lower extremity	20	T2DM, 10-20 cm^2^, >4 weeks, wound depth of Wagner grade 1 and 2 lesions	Wound closure rate, mean time to wound closure	None

### Clinical Indications and Usage of ASCs

Autologous ASCs are used in the clinical treatment of diabetic wounds and exhibited well-treating effects on patients. As the function of endogenic ASCs around the wounds is being damaged in diabetic patients, fresh ASCs generated from healthy tissues are needed for seriously damaged diabetic wounds. The indication of ASCs in clinical generation includes active diabetes with a nonhealing ischemic ulcer which approaches amputation (64, 65, 67). Furthermore, ASCs are indicated to be best-performed in wounds of Wagner Grade 2 (67). Besides, full-thickness neuropathic ulcers also respond well to ASCs treatment, indicating the function on neuro-nurturing of ASCs (66). The treating area can range from 1cm^2^ to 25cm^2^, which makes this treatment used flexibly in various wounds (66). For the usage of SVFs, a one-time injection yields SVFs ranging from 1.7 to 6.7×10^7^ in present studies (64, 65, 67, 68). The dose of injection can be flexibly adjusted in different wounds. Allogenic purified ASCs are injected into the dermo-epidermal junction within 1×10^6^ cells (68). The allogeneic ASCs sheets used in clinical studies, each with 1×10^6^ cells, were directly applied as a wound dressing to the intervention group (68). Overall, ASCs treatment can be conducted on type I and type II diabetes patients with chronic untreated wounds, which exhibit better results in Wagner grade 2 wounds.

The route of ASCs clinical application is an important factor in diabetic wound treatment. The clinical use of ASCs includes direct injection, topical gel treatment, and engineered skin graft sheet. The utilization of ASCs always depends on the clinical state of wound. Previous studies showed intra-arterial and intra-muscular injection on chronic limb ischemia results in a better micro-perfusion (69). However, intra-arterial injection may not increase the efficacy of treatment because of the lack of perfusion in diabetic peri-wound area (70). As for the clinical application of ASCs, Moon et al. and Carstens et al. injected autologous adipose-derived stromal vascular fraction (SVF) cells to diabetic patients, which contains ASCs and stromal cells essential for epidermal reconstruction (64, 65). The injection sites are designed to be around the margin of the wound (64, 65). This peri-wound injection can deliver ASCs efficiently into the treatment site. However, because injection operation requires sterility, most patients can only receive one injection (64, 65). Besides, because of the long generation period of pure ASCs, patients can only receive autologous adipose-derived stromal vascular fraction (SVF) cells injection (65). Only one clinical study reported allogenic ASCs generation on diabetic wounds (68). In patients with autologous ASCs injected, 86% of patients achieved complete closure by the end of the sixth month (64). In patients with allogenic ASCs injection, the meantime to wound closure is 31 days, which is significantly shorter than patients receiving standard treatment (68). Despite direct injection, ASCs can be delivered through biological scaffolds. Both pro-regenerative scaffolds and bioabsorbable scaffolds are used in cutaneous wound healing (71). The scaffolds can be built based on natural, synthetic and natural-synthetic hybrid materials (72). As the survival rate of ASCs is limited in traditional injection, biological scaffolds cell delivery systems provide appropriate environment for cell adhesion, proliferation, and differentiation (73, 74). In recent studies, ASCs can be delivered by nanosheets, artificial gel, artificial dermis, cell matrix derived hydrogels, silk fibroin, acellular dermal matrix based and gene-activated scaffolds (49, 52, 61, 75–83). Natural scaffolds are becoming popular because of the well biocompatibility. Animal studies have shown excellent improvement of ASCs effect on diabetic wound treatment delivered by hydrogel from human adipose tissue matrix (75). Ding et al. used collagen to deliver ASCs in diabetic mice models, indicating a stronger effect on vasculogenesis and immunomodulation (49). Silk fibroin based ASCs delivery system can improve tissue regeneration as well as survive in pathological environment (76). Enhancement of angiogenesis in subdermal tissues is detected in these studies (49, 76). ASCs can also be delivered by gene-activated scaffold, in which typical gene related to wound healing process is facilitated (52, 80). Laiva et al. used SDF-1α gene-activated collagen scaffold to contend with the pathological environment of diabetic wounds (52). Suku et al. investigated β-klotho gene-activated scaffold to reduce the release of interleukin-8 to suppress inflammatory process (80). In addition, gene-modified scaffolds may be more targeted to specific pathological conditions. The combination of ASCs and acellular dermal matrix (ADM) can also enhance wound repair in diabetic mice models, in which ADM support the proliferation and growth of ASCs (61, 77). Besides, artificial engineered scaffolds such as gelatin gel, artificial dermis, and porous polymer ultrathin films have been studied as ASCs delivering technologies (78, 79, 81, 82). A bioprinted gelatin methacryloyl hydrogel scaffolds has been shown to present a higher resistance to anoxic environment (82). This indicated that artificial scaffold delivery system may become a future choice in refractory wounds treatment, especially in diabetic wounds. Overall, natural scaffolds are superior in its well biocompatibility, while gene-activated scaffolds and artificial scaffolds can be designed more targeting on specific diabetic environment.

Animal studies have revealed that diabetic ASCs have a weakened function on diabetic wound improvement. However, none of the clinical studies have evaluated the difference between healthy ASCs and diabetic ASCs in human wound healing.

On the other hand, although previous clinical studies used subdermal injection methods, there is still a risk of vascular embolism synthesis that can aggravate the injury of diabetic wounds. However, more studies have been exploring a less traumatic administration route. Clinical studies that use topical ASCs also reported a positive result in diabetic wound healing (66, 67). Moon et al. used allogenic ASCs-hydrogel complex as the dressing over the diabetic wounds, and the dressing can be changed at first-time follow-up, presenting a method of long-time use of the treatment (65). Nilforoushzadeh et al. used an engineered skin graft containing ASCs, fibroblasts, keratinocytes, and other epidermal compositions to imitate a normal wound healing environment (66).

### Safety and Concern

Among all 158 patients reported in clinical treatments, no adverse events were reported during the follow-up periods (64–68). The common adverse events include cellulitis on unexpected sites, uncontrolled diabetic events, gangrene, and cardiac arrest. However, none of the reported adverse events were relevant to the primary diabetic wound treatment (67). Overall, no significant adverse events were reported during the most prolonged 4-year follow-up period. However, ASCs have the potential of differentiation; thus, there may be a risk of tumor in ASCs-treated patients. Therefore, whether ASCs can be used as a safe method still needs a more extended follow-up period. The recurrence was only observed in topical application of ASCs treatment (67). The recurrence sites are concentrated on regions that suffer from pressure, such as tip-top and plantar foot, and were thought to be irrelevant to the treatment (67).

### Prospects

Although the current clinical use of ASCs on diabetic wound healing achieved success, how ASCs affect the healing process remains unknown. Studies raised that ASCs promote neovascularization in diabetic wounds to accelerate diabetic wound healings (64, 67). Both studies on ACSs were clinical feasibility studies, arguing that ASCs accelerate wound healing by promoting vascularization and improving peripheral perfusion. However, due to the limit of an ethical issue, it is hard for investigators to explore the histological mechanisms. Controlled clinical trials have preliminarily shown that using ASCs may lead to a better consequence of wound healing. Further evaluations can be added to controlled clinical studies to determine the state of perfusion in peri-wound areas (68).

New metrics have emerged to be applied in evaluating the status of diabetic wounds. In Moon et al.’s study, the oxygenation and degree of perfusion were evaluated through transcutaneous partial oxygen pressure (TcPO_2_) and cutaneous microvascular blood flow (67). All ten included patients diagnosed with diabetic wounds showed significant elevations in TcPO_2_. This new technique can be used in evaluating the perfusion of diabetic wounds in future clinical trials.

The scale of present studies was small (a total of 158 patients), and most studies failed to achieve a blinded-study design. To our knowledge, allogeneic ASCs from healthy individuals are likely to have an outstanding ability in promoting diabetic wound healing, while autologous SVFs or ASCs showed mainly to improve the perforation of diabetic wounds. However, no studies have compared the efficiency between diabetic and non-diabetic ASCs. Another limitation of current studies is that the follow-up time remains limited, during which the potential risk of cancer development caused by ASCs cannot be screened.

Future studies may focus on a larger scale, double-blinded designed trials. Recent clinical studies on ASCs are all limited by the method of administration, while a feasible pattern of achieving double-blinded administration was provided. Allogeneic ASCs were gained to build a cell bank, which provides convenient storage of activated and efficient cells, and can achieve mass production. With the development of the new delivery system, more rigorous and larger scales of clinical trials should be performed in the future. Besides, ASCs sheets can achieve a long-term effect on wounds. To date, no studies have compared the efficacy of ASCs between surface medicating and injection.

## Conclusion

Previous studies approved that ASCs may act as a candidate for diabetic wound treatments with their function of autocrine, paracrine, and proliferation in the promotion of immunomodulation, neovascularization, and fibrosis. ASCs have also been tested clinically in diabetic wounds treatments. New methods of ASC delivery have been raised in recent years to provide a standardized, convenient cell bank of ASCs. More clinical trials evaluating the efficacy and safety of ASCs are needed in the future.

## Author Contributions

Conceptualization, HW; Methodology, RL; Validation, RL and RD; Formal Analysis, RL and RD; Investigation and Resources, RL, RD, MC, and XL; Writing – Original Draft Preparation, RL; Writing – Review & Editing, HW; Supervision, HW; Funding Acquisition, HW. All authors contributed to the article and approved the submitted version.

## Conflict of Interest

The authors declare that the research was conducted in the absence of any commercial or financial relationships that could be construed as a potential conflict of interest.

## Publisher’s Note

All claims expressed in this article are solely those of the authors and do not necessarily represent those of their affiliated organizations, or those of the publisher, the editors and the reviewers. Any product that may be evaluated in this article, or claim that may be made by its manufacturer, is not guaranteed or endorsed by the publisher.
